# Analysis of Polyphenolic Compounds in Extracts from Leaves of Some* Malus domestica* Cultivars: Antiradical and Antimicrobial Analysis of These Extracts

**DOI:** 10.1155/2016/6705431

**Published:** 2016-12-14

**Authors:** Alina Sowa, Grażyna Zgórka, Aleksandra Szykuła, Roman Franiczek, Beata Żbikowska, Andrzej Gamian, Zbigniew Sroka

**Affiliations:** ^1^Department of Pharmacognosy, Faculty of Pharmacy, Wrocław Medical University, Ul. Borowska 211A, Wrocław, Poland; ^2^Chair and Department of Pharmacognosy with Medicinal Plant Units, Faculty of Pharmacy with Medical Analytics Division, Medical University of Lublin, Ul. Chodźki 1, Lublin, Poland; ^3^Department of Microbiology, Faculty of Medicine, Wrocław Medical University, Ul. Chałubińskiego 4, Wrocław, Poland; ^4^Department of Medical Biochemistry, Faculty of Medicine, Wrocław Medical University, Ul. Chałubińskiego 10, Wrocław, Poland

## Abstract

In this study, methanol, ethyl acetate, water extracts, and precipitate were obtained from leaves of* Malus domestica* cultivars: Golden delicious, Jonagold, Elstar, Ligol, and Mutsu. Antiradical activity of these extracts was measured using the ABTS^+∙^ radical, and antimicrobial activity was measured with the disk-diffusion method. Phenolic compounds were measured with the colorimetric method and identified with high performance liquid chromatography (HPLC). The highest antiradical activity was observed for the Jonagold variety, and in particular strong activity was noted for ethyl acetate extracts. Antimicrobial activity was observed against strains of* Staphylococcus aureus*,* Enterococcus faecalis*, and the fungus* Candida glabrata*. Particularly susceptible to the extracts activity appeared to be* Staphylococcus aureus*, but the growth of* Candida glabrata* was inhibited in the presence of ethyl acetate extracts. With the HPLC method we identified a high amount of phloridzin (above 500 mg per g of ethyl acetate extracts), lower amounts of hyperoside, isoquercitrin, and quercitrin, and traces of* p*-hydroxybenzoic and chlorogenic acids. The contribution of phloridzin to antiradical activity of methanol and ethyl acetate extracts was very high (above 90%). In water extract the contribution of phloridzin was between 38.9 and 55.2%, chlorogenic acid 22.7 and 36.1%, and hyperoside 12.2 and 13.3%.

## 1. Introduction

Apple (*Malus domestica*) is one of the most widely cultivated trees, whose fruits are a very popular foodstuff [1, 2]. The composition of phenolic compounds present in apple fruits and their antioxidative properties have been extensively investigated [3–5]. Little is known about the composition of phenolic compounds in leaves, which are waste products that could be a valuable raw material for the preparation of extracts or phenolic antioxidants. Obtained active fractions could be used for the production of cosmetics and dietary supplements with strong antioxidant activity [6–8].

The composition of phenolic compounds present in the leaves of apple has been described in several papers [9–11]. Among polyphenolic compounds, the most frequently mentioned are flavonoids such as phloretin and its glycosides, quercetin and its glycosides (rutin, avicularin, and quercitrin), kaempferol and its glycosides, apigenin, and luteolin and its glycosides [12]. There have also been identified phenolic acids such as chlorogenic acid, caffeic acid derivatives,* p*-coumaric acid derivatives, epicatechin, and procyanidin B2 [9, 13, 14].

Phenolic compounds eliminate free radicals and do not allow for their formation [15] and thus protect the body against damage. Polyphenolic compounds derived from plants thanks to their antioxidant activity exhibit many medicinal properties such as anti-inflammatory [16], anticancer [17], antiatherogenic [18], antihemorrhagic [19], antibacterial [20], antifungal [21], antiviral [22], spasmolytic [23], diuretic [24], choleretic [25], and hepatoprotective [26] activity. Extracts from apple leaves are rich in phloridzin, which exhibit antidiabetic activity [27].

The aim of this work was to obtain different extracts from leaves of some cultivars of the species* Malus domestica* Borkh., determine the phenolic compounds, and measure the antiradical and antimicrobial activity of these extracts.

## 2. Materials and Methods

### 2.1. Raw Material

Leaves of* Malus domestica* Borkh., varieties Golden delicious, Jonagold, Elstar, Ligol, and Mutsu, were collected in October 2013 in the experimental orchard of Wrocław University of Environmental and Life Sciences. The voucher specimen is deposited in the Department of Horticulture, Wrocław University of Environmental and Life Sciences. Raw material was dried in the shade and air circulation at room temperature.

### 2.2. Preparation of Extracts

**Figure 1:**
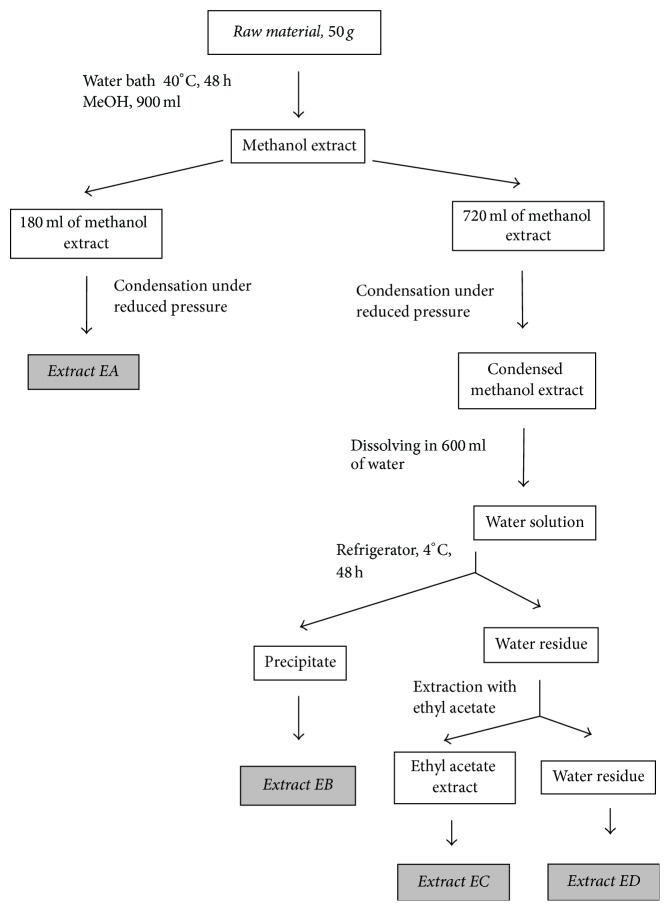
Scheme of preparation of extracts EA, EB, EC, and ED.

Scheme of preparation of extracts was presented in scheme (Figure 1). Fifty grams of ground raw material was extracted with 900 ml of methanol (POCh, Poland) at 40°C, for two days. Then the extract was filtered with filter paper (Chempur, Poland). Twenty percent (180 ml) of total extract volume (900 ml) was separated and condensed under reduced pressure to obtain dry extract EA. The remaining methanol extract was condensed under reduced pressure and the dry residue was dissolved in water at 70°C. Aqueous solution, after cooling to room temperature, was placed in the refrigerator for 24 h (4°C). The formed precipitate was separated with paper filter (Filtrak 288, Th. Wiesenbad, Germany) and dried to obtain dry extract EB. The remaining solution after precipitate separation was extracted with the following volumes of ethyl acetate (POCh): 3 × 200 ml, 4 × 100 ml. The combined ethyl acetate extracts were evaporated under reduced pressure to obtain dry extract EC. The aqueous residue was condensed under reduced pressure to obtain dry residue ED. Additionally, extracts were marked with a lowercase letter, depending on the raw material. Extracts from “Golden delicious” were marked with “g,” extracts from “Jonagold” with “j,” extracts from “Elstar” with “e,” extracts from “Ligol” with “l,” and extracts from “Mutsu” with “m.” The weight of the extracts is shown in Table 1.

### 2.3. Measurement of Total Amount of Phenolic Compounds

The amount of phenolic compounds in extracts was measured with the method of Singleton and Rossi [28], described later by Bahorun et al. [29] with some modification. 0.5 ml of Folin-Ciocalteu's phenol reagent (Na_2_WO_4_/Na_2_MoO_4_) (Sigma-Aldrich, USA) was added to the test tube; then 0.5 ml of investigated extract was added at the following concentrations: EA and EC at 0.6 mg/ml and EB and ED at 2.9 mg/ml. After 2 minutes, 2 ml of 1.89 mol/dm^3^ water solution of sodium carbonate (Na_2_CO_3_) was added to the reaction mixture and samples were heated in a boiling water bath for 1 min. After cooling, absorbance of samples was measured (Cecil 3021 spectrophotometer, Cambridge, United Kingdom) at 685 nm. Measurement was repeated five times and the maximal error was calculated. The amount of phenolic compounds was expressed as mg of gallic acid per g of extract (mgGAE/g).

### 2.4. Measurement of Antiradical Activity of Extracts

 Antiradical activity of extracts was measured according to the method of Re et al. [30]. ABTS (2,2′-azino-bis(3-ethylbenzothiazoline-6-sulfonic acid) diammonium salt, Sigma-Aldrich, USA) was dissolved in water at the concentration of 7 mmol/liter. The ABTS solution was mixed with an equal volume of 2.45 mmol/liter aqueous solution of potassium persulfate (K_2_S_2_O_8_, Sigma-Aldrich, USA). This solution was left in a dark place for 16 hours. During this time the radical cation ABTS^∙+^ was formed.

The ABTS^∙+^ solution was diluted so that absorbance was equal to 1 at *λ* = 734 nm. As a blank sample, methanol was used.

Extracts EA and EC due to high activity for all tested species were prepared at the concentration of 0.6 mg/ml, extracts EB and ED at 2.9 mg/ml.

Into each test tube 2 ml of ABTS^∙+^ in methanol was poured and the sample was preincubated at 25°C for 5 min. Then 20 *μ*l of extract solution was added. The reaction was carried out at the temperature of 25°C and the absorbance was measured (Cecil 3021) after 1 minute at 734 nm in a glass cuvette with a 1 cm optical path. A control sample was prepared by the addition of 20 *μ*l of methanol instead of the sample to the ABTS^∙+^ solution. The measurement for each extract was repeated six times.

Antiradical activity was shown as the number of antiradical units (TAU_734/mg_) per mg of extract and calculated according to (2) given below.

One unit of antiradical activity is the quantity of substance (extract) that scavenges 1 *μ*mole of ABTS^∙+^ in reaction mixture during 1 minute of reaction at 25°C.

In order to derive the following equations, we used the Beer-Lambert law *A* = *εlc* with the molar extinction coefficient of ABTS^+∙^ at 734 nm (1.5 × 10^4^ dm^3^ mole^−1^ cm^−1^) taken from the literature [30]. The decrease of concentration of ABTS^+∙^ during the reaction is associated with a decrease of absorbance as follows: Δ*c* = Δ*A*/*εl*. This relation was the basis to derive the equations:

(1) $$\begin{array}{c} {TAU}_{734/mg}=6.7\cdot 10^{-2}\frac{\left(A_{S0}-A_{S1}\right)-\left(A_{C0}-A_{C1}\right)}{c}, \end{array}$$

Because *A*_*C*0_ − *A*_*C*1_ was always zero the equation was simplified to

(2) $$\begin{array}{c} {TAU}_{734/mg}=6.7\cdot 10^{-2}\frac{\left(A_{S0}-A_{S1}\right)}{c}, \end{array}$$

where TAU_734/mg_ is the number of antiradical units per mg of substance (extract), *A*_*S*0_ is absorbance of ABTS^∙+^ solution at the beginning of the reaction, *A*_*S*1_ is the absorbance of the sample after 1 minute of reaction, *A*_*C*0_ is the absorbance of the control sample at the beginning of the reaction, *A*_*C*1_ is absorbance of the control sample after 1 minute of the reaction, and *c* is concentration of the extract in the reaction mixture [mg/ml].

The number of antiradical units was also calculated per 1 g of raw material according to the equation:

(3) $$\begin{array}{c} {TAU}_{734/g}=\frac{\left({TAU}_{734/mgEA}\cdot m_{EA}\right)+\left({TAU}_{734/mgEB}\cdot m_{EB}\right)+\left({TAU}_{734/mgEC}\cdot m_{EC}\right)+\left({TAU}_{734/mgED}\cdot m_{ED}\right)}{W_{R}}, \end{array}$$

where TAU_734/g_ is the number of antiradical units calculated per g of raw material; TAU_734/mgEA_ is the number of antiradical units per mg of EA extract; *m*_EA_ is whole mass of EA extract obtained from raw material [mg]; TAU_734/mgEB_ is the number of antiradical units per mg of EB extract; *m*_EB_ is whole mass of EB extract [mg]; TAU_734/mgEC_ is the number of antiradical units per mg of EC extract; *m*_EC_ is whole mass of EC extract [mg]; TAU_734/mgED_ is the number of antiradical units per mg of ED extract; *m*_ED_ is whole mass of ED extract [mg]; *W*_*R*_ is mass of raw material [g] taken for extraction. The maximal error was calculated with the total differential method.

### 2.5. Measurement of Antimicrobial Activity of Extracts

#### 2.5.1. Bacterial and Fungal Strains

The antimicrobial activities of the plant extracts were determined against the following bacterial and fungal strains:* Staphylococcus aureus* (ATCC 25923),* Enterococcus faecalis* (ATCC 29212),* Escherichia coli* (ATCC 25922),* Bacillus subtilis* (ATCC 6633),* Klebsiella pneumonia* (ATCC 700603),* Pseudomonas aeruginosa* (ATCC 27853), and* Candida albicans* (ATCC 90028),* Candida glabrata* (ATCC 90030), and* Saccharomyces cerevisiae* (BCMM 3963).

These strains were obtained from the Department of Microbiology, Wroclaw Medical University, Poland.

#### 2.5.2. Description of the Method

The antimicrobial activity of extracts was measured with the method described by Ingolfsdottir et al. [31].

The activity was determined by the disk-diffusion method on Mueller-Hinton agar plates (Oxoid, Basingstoke, UK) for the bacterial strains and on Sabouraud agar plates (Biomed, Lublin, Poland). The bacterial and fungal suspensions of the strains tested, at a turbidity comparable to that of 0.5 McFarland standard, were diluted in saline (1 : 10) to obtain a final inoculum of 10^7^ CFU/ml. After that, the suspensions were spread uniformly on agar plates using sterile swabs. The extract was dissolved in dimethyl sulfoxide (DMSO, Sigma-Aldrich, Poland) to obtain the concentration of 100 mg/ml. Then the standard disks (6 mm in diameter, Becton Dickinson, Sparks, USA) were placed aseptically on the agar plates.

After 15 minutes, a 10 *μ*l aliquot of DMSO-dissolved extract was placed on the disks. The plates thus prepared were incubated at 37°C for 24 or 48 hours for the bacterial and fungal strains, respectively. After incubation, the results were recorded by measuring the zones (mm) of growth inhibition around the disks. Ampicillin (10 *μ*g; Oxoid, Basingstoke, UK) and amphotericin B (10 *μ*g; Abtek Biologicals Ltd., Liverpool, UK) disks were used as a positive control, whereas DMSO served as a negative control. These experiments were performed in triplicate, and the results were the average of three independent experiments. Standard uncertainty was established as ±0.33 for all measurements.

### 2.6. HPLC Analysis

#### 2.6.1. Solvents, Reagents, and Materials

HPLC-grade solvents (acetonitrile, phosphoric acid) were purchased from J. T. Baker (Deventer, The Netherlands). Methanol (MeOH) of analytical grade was obtained from Avantor Performance Materials (Gliwice, Poland). Additionally, in HPLC analysis, highly purified water produced by a Direct-Q Water Purification System (Millipore, Molsheim, France) and polyphenolic reference substances, including* p*-hydroxybenzoic and chlorogenic acids obtained from Sigma-Aldrich (Buchs, Switzerland), phloridzin rutoside and isoquercitrin purchased from ChromaDex (Santa Ana, CA, USA), hyperoside (Fluka, Buchs, Switzerland), and quercitrin obtained from HWI Analytic GmbH (Ruelzheim, Germany) were used. For solid phase extraction (SPE), Phenyl BakerBond cartridges (500 mg, 3 ml) were supplied by J. T. Baker (Phillipsburg, NJ, USA).

#### 2.6.2. Preliminary Sample Clean-Up Using SPE

To remove chlorophyll and other ballast hydrophobic components present in apple leaf extracts, the SPE procedure was employed using a 12-port vacuum manifold processor (system Baker SPE-12G) connected with a vacuum pump (AGA-Labor, Warsaw, Poland). Adequate amounts of dried apple leaf extracts were taken up in 75% MeOH and transferred into phenyl J. T. Baker cartridges, activated previously with MeOH (10 ml), followed by equilibration with 75% (v/v) MeOH (10 ml). Extract samples were passed through the bed of phenyl adsorbent under vacuum (−0.01 MPa) into 10 mL calibrated flasks. Before HPLC analysis, eluates were filtered using 0.45-*μ*m Minisart SRP 15 filters (Sartorius, Goettingen, Germany).

### 2.7. HPLC Protocol

An Agilent Technologies (Waldbronn, Germany) Model 1100 liquid chromatograph, equipped with a PDA detector, a Rheodyne 20 *μ*l loop injector (10 *μ*l samples were injected), a quaternary pump, and an online vacuum degasser, was used for the separation, identification, and quantification of phenolic compounds in apple leaf extracts. The separation of polyphenolic compounds was performed on a Thermo Scientific Aquasil C18 column (250 × 4.6 mm I.D.; *d*_*p*_ = 5 *μ*m). A gradient mobile phase system with acetonitrile (A) and 1 mM phosphoric acid (B) was used as follows: 0 min/15% (A in B); 15 min/15; 25 min/20; 35 min/20; 50 min/50; and 60 min/90% A in B. The post time of 15 min was applied for the column equilibration. The flow rate was 1.0 ml min^−1^. The temperature of the thermostated column compartment was maintained at 25°C during each chromatographic separation.

The identification of the peaks on chromatograms was achieved by comparing their retention times and UV spectra (collected in the range from 190 to 400 nm) with those obtained for reference compounds.

Quantitative analysis of identified polyphenolic constituents was performed based on an external standard method. The linearity of calibration curves, obtained for particular polyphenolic compounds (Table 2), was assessed using regression coefficients (*R* ^2^) estimated for six-point curves constructed using reference compound solutions prepared in MeOH at mean working concentrations ranging from 0.02 to 0.15 mg/ml. The repeatability of peak areas was controlled by relative standard deviation (RSD) evaluation (not higher than 3%) obtained in intra- and interday (*n* = 3) assays. Flavonols (hyperoside, quercitrin, isoquercitrin, and rutoside) and* p-*hydroxybenzoic acid were quantified at 254 nm, phloridzin at 280 nm, and chlorogenic acid at 320 nm. Mean concentrations of all polyphenolic compounds were determined for extract samples obtained in triplicate, and SD and RSD values were calculated.

### 2.8. Contribution of Each Phenolic Compound to General Antioxidant Activity of Extracts (C%)

The contribution of each phenolic compound in extracts (*C*%) to the general antiradical activity of extract was calculated taking into account the activity of each compound (TAU_734/mg_) and the content of the compound in the extract (mg/g). The contribution *C*% was calculated according to the equation:

(4) $$\begin{array}{c} C\%=\frac{TAU_{734/mgx}\cdot c_{x}}{\sum_{x=Ch,H,Iq,Q,R,Ph}{TAU}_{mgx}\cdot c_{x}}\cdot 100, \end{array}$$

where *C*% is the contribution of each compound to the general activity of the extract, TAU_734/mg*x*_ is the antioxidant activity of compound *x*, *c*_*x*_ is the amount of compound *x* in the extract [mg/g], Ch is chlorogenic acid, H is hyperoside, Iq is isoquercitrin, Q is quercitrin, R is rutin, and Ph is phloridzin.

### 2.9. Statistical Analysis

The statistical analysis was performed using the *t*-test for independent samples.

## 3. Results and Discussion

Plant organs such as leaves are always rich in phenolic compounds which are beneficial for health [32, 33]. In the case of apple, one of the most widely cultivated fruit trees in the world, leaves are potentially a rich source of phenolic compounds with strong antiradical as well as antimicrobial activity.

Chemical composition, especially the presence of phenolic compounds, has been widely investigated in fruits [13], but there is much less information related to content of phenols in leaves of apple [9]. The intensive research on the phenolic compounds present in apple leaves may lead to the use of this raw material as a medicine.

### 3.1. Determination of Total Phenolic Compounds and Antiradical Activity of Extracts

The amount of phenolic compounds in extracts measured by the colorimetric method and antiradical activity is presented in Table 1. A high amount of phenolic compounds was noted for EC extracts, the highest for ethyl acetate extracts obtained from “Mutsu” and “Ligol” variety, respectively, 329 ± 4 and 328 ± 4 mg of phenolic compounds per g of extract expressed as gallic acid (mgGAE/g). A low amount of total phenols was measured for EB and ED extracts, the lowest for extracts ED_e_ and ED_m_, respectively, 41.2 ± 0.6 and 32.1 ± 0.2 mgGAE/g.

As in the content of phenolic compounds, the high antiradical activity TAU_734/mg_ was observed for extracts EC and a little lower for extracts EA. The highest antiradical activity (TAU_734/mg_) was observed for extract EC_j_ from the “Jonagold” (4.68 ± 0.22) and extract EC_l_ from the “Ligol” (4.4 ± 0.12). Low antiradical activity (TAU_734/mg_) was detected for extracts EB and ED, the lowest for EB_g_ and ED_e_ extracts from “Golden delicious” and “Elstar,” respectively, 0.64 ± 0.05 and 0.60 ± 0.03.

**Figure 2:**
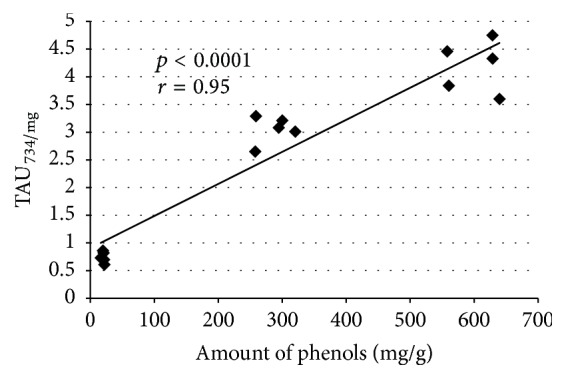
Pearson's correlation coefficient (*r*) between number of antiradical units per mg of extract (TAU_734/mg_) and amount of phenolic compounds (measured with HPLC) expressed in mg per gram of extract.

There was observed strong positive correlation (*r* = 0.95, Figure 2) between number of antiradical units per mg of extract and amount of phenols (sum of phenols determined with HPLC) expressed in mg of phenols per g of extract.

**Figure 3:**
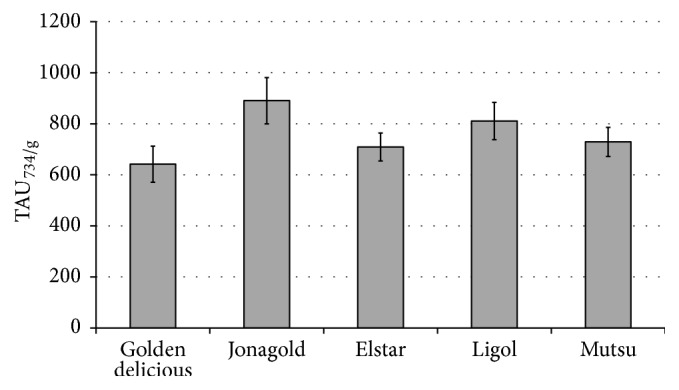
Number of antiradical units per g of raw material (TAU_734/g_) for cultivars: Golden delicious, Jonagold, Elstar, Ligol, and Mutsu. Maximal errors are marked with the bars (*n* = 6).

The number of antiradical units was also calculated per g of raw material (Figure 3). The highest number of antiradical units was calculated per g of “Jonagold,” the value of TAU_734/g_ being 877 ± 79, but the lowest for “Golden delicious,” the amount of antiradical units per g of raw material (TAU_734/g_) being 628 ± 71.

HPLC analysis showed that the main phenolic fractions were flavonoids, with the highest amount of phloridzin. Similar results were described by Liaudanskas et al. [9].

The antiradical features of raw materials (TAU_734/g_) are similar to the results obtained for white and green tea leaves by Wojciechowski et al. [34].

### 3.2. Antimicrobial Activity of Extracts

The inhibition of growth of bacteria by the extracts from apple leaves is demonstrated in Table 3. Activity of extracts was studied against strains given in section bacterial and fungal strains. Growth inhibitory activity was observed only against two gram positive bacterial strains* Staphylococcus aureus* ATCC 25923 and* Enterococcus faecalis* ATCC 29212 and against the strain of fungus* Candida glabrata* ATCC 90030.

**Figure 4:**
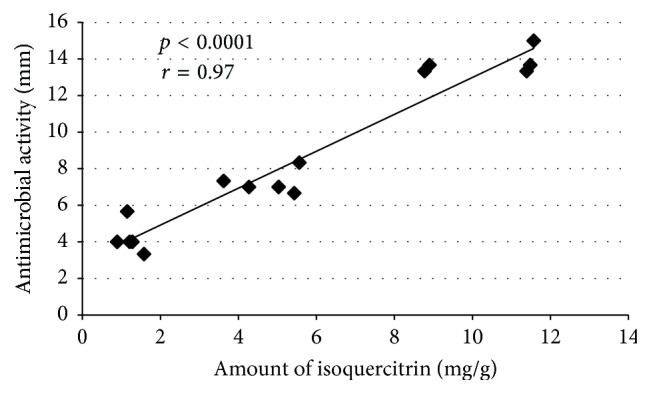
Pearson's correlation coefficient (*r*) between antimicrobial activity of extracts expressed as diameter of zone of growth inhibition (mm) and amount of isoquercitrin (measured with HPLC) expressed in mg per gram of extract.

High activity was observed for EC extracts but the highest for EC_l_ extract from “Ligol” leaves against* Staphylococcus aureus* (19 ± 0.33). The highest activity against* Enterococcus faecalis* was exhibited by ED_e_ (17 ± 0.33), with a relatively small amount of phenolic compounds (4.12 ± 0.6). It is interesting that extracts ED with relatively low antibacterial activity were active against* Enterococcus faecalis*. These extracts are different from others due to the relatively high content of rutin and chlorogenic acid (Table 4), which is a strong antimicrobial compound. There was observed positive correlation (*r* = 0.97) between the amount of isoquercitrin in extracts and antimicrobial activity of these extracts (Figure 4).

Phloridzin, chlorogenic acid, and other flavonoids exhibit antimicrobial activity [35, 36]. The general antimicrobial effect depends on the concentration of the substance and sensitivity of the microorganism to this substance. The concentration of phenols in our extracts is sufficient to show antibacterial activity.

### 3.3. Analysis of Phenolic Compounds with High Performance Liquid Chromatography

Due to low yield and weak antiradical activity of extracts EB (Table 1) they are not an interesting potential source of phenolic compounds. The analysis of phenolic compounds with HPLC was conducted only for extracts EA, EC, and ED.

The linear regression data for HPLC analysis of polyphenols identified in extracts is shown in Table 2.

The results of analysis of compounds present in extract EA are shown in Table 5. After summarizing the amount of compounds identified in EA extracts for all cultivars, the highest amount of phenols expressed as mg per g of extract was determined for the “Ligol” variety (320 ± 4.1) and the lowest for the “Mutsu” variety (258 ± 4.6). The dominating phenolic compound in EA extracts of all cultivars was phloridzin (dihydrochalcone). The highest amount of phloridzin was noted for extract EA_l_ from “Ligol” (299 ± 3.4 mg/g). Also there was noted significant amount of hyperoside (quercetin 3-O-galactoside): 10.3 ± 0.32 mg/g.

**Figure 5:**
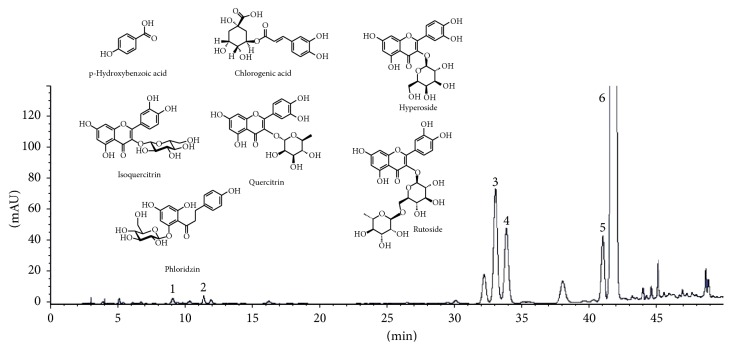
HPLC separation of polyphenolic compounds identified in ethyl acetate extract (EC_g_) obtained from the leaves of Golden apple cultivar. Compounds: 1 = chlorogenic acid; 2 =* p*-hydroxybenzoic acid; 3 = hyperoside; 4 = isoquercitrin; 5 = quercitrin; 6 = phloridzin. The structure of compounds identified in extracts (rutoside was identified only in extracts ED, Table 4).

When the EC extracts were tested (Table 6), the highest sum of phenols was observed for the “Golden delicious” cultivar (640 ± 21.9 mg/g) and lowest for “Ligol” (558 ± 19). Among phenolic compounds, phloridzin dominated in the extract from “Golden delicious” (603 ± 20.8; Figure 5). There was also noted large amount of hyperoside in extracts from “Ligol" (21.4 ± 0.60 mg/g).

ED extracts contained the lowest amount of sum of phenolic compounds (Table 4), but these extracts have some amount of rutin, unlike other extracts. The highest sum of phenolic compounds was observed for “Elstar” (22 ± 0.57 mg/g), the lowest for “Mutsu” (16.3 ± 0.31). As in the case of other extracts, in the ED extracts phloridzin was predominant, with the maximal value for “Jonagold” (13.5 ± 0.27); the highest amount of rutin was noted for extract ED_l_ from “Ligol” (1.98 ± 0.05 mg/g).

With the HPLC method there were identified mainly flavonoids but in smaller amounts* p*-hydroxybenzoic and chlorogenic acids. Among flavonoids there were identified rutin, hyperoside, isoquercitrin, quercitrin, and phloridzin (the highest amount).

Liaudanskas et al. [9] performed similar research using raw material from the experimental orchard of the Institute of Horticulture, Lithuanian Research Centre for Agricultural and Forestry, Babtai, Lithuania. They prepared 70% ethanol extracts from lyophilized leaf powder. In extracts, they were identified: hyperoside, isoquercitrin, rutin, avicularin, quercetin, phloridzin, and in less amounts chlorogenic and caffeic acid, (+)-catechin, and (−)-epicatechin.

### 3.4. Contribution (C%) of Antiradical Activity of Each Compound to Antiradical Activity of Extracts

The antiradical activity (TAU_734/mg_) of each compound was as follows: chlorogenic acid = 6.6 ± 0.1, hyperoside = 4.2 ± 0.01, isoquercitrin = 3.9 ± 0.01, quercetin = 4.5 ± 0.01, phloridzin = 3.2 ± 0.1, and rutin = 2.5 ± 0.2.

The contribution of compounds (*C*%) in extracts antiradical activity was calculated (4) including the activity and the amount of compounds (measured with HPLC). Due to very low amount and weak antiradical activity pOHBA was not taken into consideration.

The contribution to antiradical activity of compounds (*C*%) is presented in Table 7. The highest *C*% value was observed for phloridzin for all extracts, with the highest value for extract EC_m_ from the “Mutsu” cultivar, at 93.9%. An important effect was also observed for chlorogenic acid for ED extracts, with the highest value for the ED_l_ extract from “Ligol” (36.1%). A high contribution was also observed for hyperoside in ED_j_ and ED_e_ extracts from “Jonagold” and “Elstar” cultivars (13.3%). Some authors confirm significant antioxidant features of phloridzin [37, 38]. Our research demonstrated that the antiradical activity of phloridzin is high: TAU_734/mg_ = 3.2 ± 0.15 (Trolox = 5.5 ± 0.1). Phloridzin is the main antioxidant identified in extracts obtained from leaves of different cultivars of apple, and its participation in antiradical activity of extracts exceeds 90%.

Phloridzin is considered by many authors as a weak antioxidant. They believe that the structure of this compound is disadvantageous for antiradical activity [39]. Our research clearly showed that phloridzin antiradical activity is high (higher than rutin). Our earlier research [40] showed that the presence of a hydroxyl group situated at the 4′ position in the B ring is very important for antiradical activity of flavones and flavonols (see phloridzin in Figure 5). It seems that in addition to the structure of 2,4,6 trihydroxyacetophenone with a hydroxyl group bonded to aromatic ring A at position 2 in phloridzin [39], also the 4′ hydroxyl group present in the B ring may be important for antioxidant activity. Because of its strong antiradical activity and high content in extracts, the contribution of phloridzin to antiradical activity of extracts is the highest in all extracts.

### 3.5. Statistical Analysis

The statistical significance of differences between TAU_734/mg_ and TAU_734/g_ was calculated using the *t*-test for independent samples. The differences between samples were considered statistically significant when the *p* value ≤ 0.05. When TAU_734/mg_ of all extracts was analysed the *p* value was lower than 0.05 for about half of samples. The highest *p* value was calculated between EA_e_, EA_j_ (*p* = 0.85) and EB_j_, ED_m_ (*p* = 0.79) extracts. The lowest *p* value was observed for EC_l_, ED_m_ (9.81·10^−11^) and EC_l_, EB_m_ (8.63·10^−11^). The statistical significance of differences between TAU_734/g_ for raw materials is shown in Table 8. Statistical significance was demonstrated for the raw materials “Golden delicious” and “Jonagold” (*p* = 0.041).

## 4. Conclusion

Leaves of apple are rich in phenolic compounds especially flavonoids. The richest in phenolic compounds is extract EC (ethyl acetate) and extract EA (methanol) contains average amount of phenolic compounds, but extract EBs and ED were the poorest. In extracts EA and EC main phenolic fraction was flavonoids. Phloridzin was a dominating compound; quercetin glycosides such as hyperoside or isoquercitrin were in a lower amount. Phenolic acids such as p-hydroxybenzoic and chlorogenic acid were present in traces. Extract ED contains rutin, relatively less phloridzin, and more chlorogenic acid.

The highest antiradical activity was observed for extracts EC, a little lower for extracts EA. Extracts EB and ED exhibited much weaker antiradical activity. The antiradical activity was strongly positively correlated with the amount of phenols. Based on the amount of phenols and antiradical of each compound, the participation of each compound in the activity of extracts was calculated. In extracts EA and EC the highest contribution in antiradical activity was calculated for phloridzin. In ED extract contribution of phloridzin was lower but higher participation was calculated for chlorogenic acid, hyperoside, and isoquercitrin.

The strongest antibacterial activity was observed for EC extracts a little lower for EA extracts against strains of* Staphylococcus aureus* ATCC 25923 and* Enterococcus faecalis* ATCC 29212. Antifungal activity was observed only for EC extracts against* Candida glabrata* ATCC 90030.

## Figures and Tables

**Table 1:** Extracts from apple cultivars: symbols of extracts, mass of extracts, the content of phenolics and the antiradical activity of extracts (TAU_734/mg_), and raw materials (TAU_734/g_). TAU_734/mg_ value for Trolox is 5.52 ± 0.1. Maximal error was calculated by the total differential method *n* ≥ 5.

Cultivar	Symbol of extract	Weight of extract [g ± 0.0001]	Content of phenols [mgGAE/g]	TAU_734/mg_	TAU_734/g_
Golden delicious	EA_g_	2.7597	114 ± 2	3.03 ± 0.5	628 ± 71
	EB_g_	1.2262	73 ± 1	0.64 ± 0.05	
	EC_g_	4.8471	274 ± 4	3.5 ± 0.24	
	ED_g_	6.2138	45 ± 1	0.85 ± 0.05	

Jonagold	EA_j_	3.2726	151 ± 2	3.16 ± 0.41	877 ± 79
	EB_j_	1.3051	65 ± 1	0.74 ± 0.04	
	EC_j_	5.9319	255 ± 7	4.68 ± 0.22	
	ED_j_	6.9537	45 ± 1	0.69 ± 0.05	

Elstar	EA_e_	3.1866	144 ± 2	3.24 ± 0.23	699 ± 54
	EB_e_	1.1381	42 ± 0.6	0.69 ± 0.04	
	EC_e_	5.1243	249 ± 7	3.79 ± 0.19	
	ED_e_	7.3648	41.2 ± 0.6	0.60 ± 0.03	

Ligol	EA_l_	3.2210	146 ± 2	2.97 ± 0.56	800 ± 73
	EB_l_	1.1750	121 ± 2	1.14 ± 0.08	
	EC_l_	5.4584	328 ± 4	4.4 ± 0.12	
	ED_l_	6.2576	44 ± 0.6	0.81 ± 0.04	

Mutsu	EA_m_	3.2771	120 ± 1.2	2.61 ± 0.2	729 ± 57
	EB_m_	1.3650	87.5 ± 1.2	0.97 ± 0.03	
	EC_m_	4.8193	329 ± 4	4.27 ± 0.2	
	ED_m_	7.6102	32.1 ± 0.2	0.72 ± 0.06	

**Table 2:** Linear regression data (*y* = *ax* + *b*): slope (*a*), intercept (*b*), and regression coefficient (*R* ^2^) for polyphenolic compounds identified in extracts obtained from leaves of various apple cultivars.

Compound	*a*	*b*	*R* ^2^
P-Hydroxybenzoic acid	60859.4	−6.0	0.99997
Chlorogenic acid	29208.3	7.0	0.99998
Hyperoside	16480.0	−2.6	0.99998
Isoquercitrin	18098.1	2.6	0.99996
Quercitrin	25047.7	3.3	0.99997
Rutoside	12657.9	−4.6	0.99996
Phloridzin	19560.9	3.7	0.99998

**Table 3:** Antimicrobial activity of extracts from leaves of different varieties of apple demonstrated as a diameter of inhibition zone of bacterial or fungal growth [mm]. Antimicrobial activity was observed against three out of 9 strains tested: two gram positive bacterial (*Staphylococcus aureus*, *Enterococcus faecalis*) and fungal (*Candida glabrata*) strains. Statistical uncertainty was equal to ±0.33 (*n* = 3).

Cultivar	Extract	*Staphylococcus aureus* ATCC 25923	*Enterococcus faecalis* ATCC 29212	*Candida glabrata* ATCC 90030
Ligol	EA_l_	13	12	0
	EB_l_	10	0	0
	EC_l_	19	11	15
	ED_l_	0	10	0

Golden delicious	EA_g_	10	11	0
	EB_g_	8	0	0
	EC_g_	15	15	10
	ED_g_	0	12	0

Elstar	EA_e_	11	10	0
	EB_e_	8	0	0
	EC_e_	16	13	12
	ED_e_	0	17	0

Jonagold	EA_j_	10	10	0
	EB_j_	8	0	0
	EC_j_	15	11	15
	ED_j_	0	12	0

Mutsu	EA_m_	11	11	0
	EB_m_	10	0	0
	EC_m_	17	13	10
	ED_m_	0	12	0

Ampicillin (10 *µ*g/disc)		32	22	—

Amphotericin B (10 *µ*g/disc)		—	—	15

0: no inhibition observed.

—: activity not investigated.

**Table 4:** Mean (*n* = 3) content, expressed in mg/g dry wt ± SD (RSD, %), of polyphenolic compounds identified in aqueous (ED) extracts obtained from the leaves of five *Malus* Mill. cultivars.

Cultivar	Polyphenols							
	pOHBA	ChlorA	Hyper	Iquer	Quer	Rut	Phlor	Sum
Elstar	0.14 ± 0.003 (2.5)	4.35 ± 0.15 (3.5)	2.79 ± 0.05 (1.9)	1.58 ± 0.05 (3.0)	0.10 ± 0.002 (2.4)	1.70 ± 0.06 (3.3)	11.26 ± 0.26 (2.3)	22.02 ± 0.57
Golden delicious	0.13 ± 0.003 (2.6)	3.26 ± 0.04 (1.1)	2.25 ± 0.05 (2.4)	1.28 ± 0.04 (3.2)	0.17 ± 0.01 (3.1)	0.77 ± 0.02 (2.8)	11.79 ± 0.22 (1.9)	19.65 ± 0.38
Jonagold	0.09 ± 0.003 (2.9)	3.42 ± 0.06 (1.8)	2.39 ± 0.06 (2.4)	1.15 ± 0.04 (3.5)	0.13 ± 0.05 (3.6)	0.99 ± 0.02 (2.2)	13.47 ± 0.27 (2.0)	21.64 ± 0.50
Ligol	0.13 ± 0.004 (3.2)	4.56 ± 0.14 (3.1)	2.61 ± 0.05 (1.8)	1.21 ± 0.03 (2.4)	0.07 ± 0.003 (3.8)	1.98 ± 0.05 (2.6)	10.08 ± 0.27 (2.7)	20.64 ± 0.55
Mutsu	0.10 ± 0.004 (3.7)	2.10 ± 0.03 (1.6)	1.79 ± 0.04 (2.0)	0.89 ± 0.03 (3.6)	0.11 ± 0.002 (1.9)	0.84 ± 0.03 (3.4)	10.48 ± 0.17 (1.6)	16.31 ± 0.31

Explanations: pOHBA: *p-*hydroxybenzoic acid; ChlorA: chlorogenic acid; Hyper: hyperoside; Iquer: isoquercitrin; Quer: quercitrin; Rut: rutoside; Phlor: phloridzin.

**Table 5:** Mean (*n* = 3) content in mg/g dry wt ± SD (RSD, %) of polyphenolic compounds identified in methanolic (EA) extracts obtained from the leaves of five *Malus* L. cultivars.

Cultivar	Polyphenols						
	pOHBA	ChlorA	Hyper	Iquer	Quer	Phlor	Sum
Elstar	0.11 ± 0.003 (2.4)	2.66 ± 0.08 (3.1)	9.24 ± 0.32 (3.4)	5.56 ± 0.16 (2.9)	2.12 ± 0.06 (3.0)	239.24 ± 6.21 (2.6)	258.93 ± 6.83
Golden delicious	0.13 ± 0.004 (2.8)	2.62 ± 0.05 (2.1)	8.24 ± 0.23 (2.9)	5.03 ± 0.15 (2.9)	2.65 ± 0.03 (1.2)	278.46 ± 2.58 (0.9)	297.13 ± 3.04
Jonagold	0.07 ± 0.002 (3.1)	2.50 ± 0.04 (1.5)	8.15 ± 0.09 (1.1)	4.27 ± 0.09 (2.2)	2.54 ± 0.03 (1.3)	282.42 ± 6.63 (2.3)	299.95 ± 6.88
Ligol	0.10 ± 0.005 (4.7)	3.08 ± 0.08 (2.7)	10.35 ± 0.32 (3.1)	5.43 ± 0.20 (3.6)	2.44 ± 0.08 (3.3)	299.00 ± 3.44 (1.2)	320.4 ± 4.12
Mutsu	0.10 ± 0.004 (4.2)	1.66 ± 0.05 (2.7)	6.70 ± 0.20 (3.0)	3.62 ± 0.07 (2.0)	1.96 ± 0.04 (2.2)	243.80 ± 4.20 (1.7)	257.8 ± 4.6

Explanations: pOHBA: *p-*hydroxybenzoic acid; ChlorA: chlorogenic acid; Hyper: hyperoside; Iquer: isoquercitrin; Quer: quercitrin; Phlor: phloridzin.

**Table 6:** Mean (*n* = 3) content in mg/g dry wt ± SD (RSD, %) of polyphenolic compounds identified in ethyl acetate (EC) extracts obtained from the leaves of five *Malus* L. cultivars.

Cultivar	Polyphenols						
	pOHBA	ChlorA	Hyper	Iquer	Quer	Phlor	Sum
Elstar	0.17 ± 0.003 (1.5)	0.90 ± 0.02 (2.1)	18.70 ± 0.47 (2.5)	11.57 ± 0.34 (2.9)	4.44 ± 0.17 (3.8)	524.7 ± 8.44 (1.6)	560.5 ± 9.44
Golden delicious	0.24 ± 0.01 (3.8)	0.81 ± 0.03 (3.7)	18.62 ± 0.61 (3.3)	11.39 ± 0.24 (2.1)	5.60 ± 0.19 (3.4)	603.1 ± 20.85 (3.5)	639.8 ± 21.93
Jonagold	0.36 ± 0.01 (1.6)	1.02 ± 0.03 (2.9)	16.47 ± 0.47 (2.8)	8.90 ± 0.17 (1.9)	5.16 ± 0.19 (3.7)	597.0 ± 18.19 (3.0)	628.9 ± 19.06
Ligol	0.12 ± 0.004 (3.6)	1.07 ± 0.03 (2.8)	21.44 ± 0.60 (2.8)	11.48 ± 0.35 (3.0)	5.20 ± 0.19 (3.7)	518.7 ± 17.83 (3.4)	558.0 ± 19.00
Mutsu	0.22 ± 0.005 (2.0)	0.61 ± 0.02 (3.2)	15.63 ± 0.52 (3.3)	8.77 ± 0.24 (2.7)	5.00 ± 0.17 (3.3)	598.8 ± 14.85 (2.5)	629.0 ± 15.80

Explanations: pOHBA: *p-*hydroxybenzoic acid; ChlorA: chlorogenic acid; Hyper: hyperoside; Iquer: isoquercitrin; Quer: quercitrin; Phlor: phloridzin.

**Table 7:** Contribution of individual phenolic compounds in general antioxidant activity of extracts expressed in percentages (*C*%).

Cultivar	Extract	Percentage contribution of compounds in antiradical activity of extracts *C*%					
		ChlorA	Hyper	Iquer	Quer	Rut	Phlor
Golden delicious	EA_g_	0.09	3.6	2.0	1.2	—	93.1
	EC_g_	0.25	3.7	2.1	1.2	—	92.7
	ED_g_	28.0	12.5	6.5	1.0	2.5	49.5

Jonagold	EA_j_	1.7	3.4	1.7	1.2	—	92.0
	EC_j_	0.33	3.3	1.7	1.1	—	93.5
	ED_j_	26.0	13.3	7.0	0.7	3.0	52.7

Elstar	EA_e_	2.0	4.5	2.5	1.1	—	89.8
	EC_e_	0.32	4,2	2.4	1.1	—	91.9
	ED_e_	32.9	13.3	7.0	0.5	4.8	41.4

Ligol	EA_l_	1.9	4.1	2.0	1.0	—	91.0
	EC_l_	0.38	4.9	2.4	1.3	—	91.0
	ED_l_	36.1	13.1	5.6	0.4	5.9	38.9

Mutsu	EA_m_	1.3	3.3	1.6	1.0	—	92.7
	EC_m_	0.19	3.18	1.6	1.1	—	93.9
	ED_m_	22.7	12.2	5.6	0.8	3.4	55.2

**Table 8:** Statistical significance of differences between TAU_734/g_ values of raw materials calculated with *t*-test for independent samples expressed as *p* value, *n* = 6.

Raw material	Golden delicious	Jonagold	Elstar	Ligol	Mutsu
Golden delicious	1	0.041	0.445	0.122	0.293
Jonagold	0.041	1	0.092	0.490	0.160
Elstar	0.445	0.092	1	0.292	0.710
Ligol	0.122	0.490	0.292	1	0.461
Mutsu	0.293	0.160	0.710	0.461	1

## Abbreviations

TAU_734/mg_: Number of antiradical units per mg of extract
TAU_734/g_: Number of antiradical units per g of raw material
EA: Methanol extract
EB: Precipitate formed during extraction
EC: Ethyl acetate extract
ED: Aqueous residue after extraction
*C*%: Participation of individual phenolic compound in antiradical activity of extracts.
